# Panoramic analysis of 2D dirubidium telluride monolayer benchmarking the DFT approach

**DOI:** 10.1038/s41598-025-89149-z

**Published:** 2025-02-07

**Authors:** G. Sneha, R. D. Eithiraj

**Affiliations:** Department of Physics, School of Advanced Sciences, Vellore Institute of Technology (VIT), 600 127, Chennai, Tamil Nadu India

**Keywords:** Two-dimensional materials, Density functional theory, Material properties, Rb_2_Te monolayer, Optoelectronic, Thermoelectric, Theory and computation, Electronic properties and materials

## Abstract

Through the DFT computations, the structural, vibrational, electronic, elastic, optical and thermal (thermoelectric, thermodynamic) properties of the two-dimensional Rb_2_Te monolayer are briefly contemplated. The Perdew-Bruke-Ernzerhof (PBE) form of generalized gradient approximation (GGA) functional in WIEN2k was deployed for the analysis of all these material properties. The trigonally crystallizing monolayer with an indirect band gap of 1.72 eV may be an upright single-layer that suffices distinct applications. ‘No negative’ phonon bands confirm the dynamical stability of the monolayer. The Rb_2_Te monolayer has large indirect band gap than Rb_2_S and Rb_2_Se. It exhibits mechanical stability with positive elastic constants satisfying the Born-Huang criterion for two-dimensional materials. The absorption coefficient spanning largely in the ultra-violet range makes the monolayer a congruous material for UV applications. Also, the thermoelectric figure of merit for *p*-type Rb_2_Te single-layer at room temperature is high (0.67) compared to the analogous series of compounds, that makes the monolayer a viable one for thermoelectric flexibility and experimental synthesis. The monolayer has high hole effective mass and D ratio. The obtained results aids in revealing the outstanding properties and excellent stability of the monolayer. Based on these findings the Rb_2_Te monolayer paves the way for promising applications in the fields of photovoltaics, thermoelectrics and UV-based applications.

## Introduction

The two-dimensional (2D) materials have been potentially revolutionized, which originated from the earliest decades of the twentieth century and are vastly grown till today. The highly debated issue was about the synthesis and stability of the 2D materials. It was achieved by the exfoliation of graphene from graphite in 2004 and by the synthesis of popular 2D molybdenum disulphide^1,2^. These two structures are broadly studied and have boundless applications. Boron nitride and TMDCs are extensively used in electronics and optoelectronics^3^. Expeditious advancements in alkali metal chalcogenides with their exceptional applications in emerging fields of research and technology have made the researchers to potentially work on this combination of compounds in the recent years. They seemingly possess large energy band gaps and have engrossing (physical and physiochemical) properties as functional materials^4^. In Astro particle physics, the especial compound in this family Rubidium Telluride is said to have profound applications as hybrid photodiode (HPD) and is used as a precursor material for producing Rb_2_Te/Te fluxes that in turn is developed as quaternary metal tellurides^5^. The theoretical studies of the rubidium telluride have been carried out by calculating the attributes like lattice constants, bulk modulus, and phase diagrams. The TB-LMTO method was used to analyze the band nature of the 3D Rb_2_Te which was reported by Eithiraj et al.^6^. The 3D Rb_2_Te is said to crystallize into a stable anti-fluorite structure (or anti-CaF_2_ structure) in the space group Fm-3 m at room temperature, but when introduced to heat or upon warming it transforms into an irreversible anti-PbCl_2_ structure type. The metastability displayed by the 3D Rb_2_Te was examined by S.M. Alay-e-Abbas et al., and A. Shaukat et al., via FP-LAPW (with the GGA exchange correlation potential). They also reported the structural, electronic, and optical nature of the polymorphic forms of 3D Rb_2_Te. The thermodynamic properties were studied and outlined by K. Bidai et al., under high pressures. The lattice dynamical and elastic attributes are reported by Z. Souadia via GGA approximation^4–7^. Lately, Shaukat Ali Khattak et al., investigated the metal-alkali-based binary chalcogenides and has disclosed that the 3D Rb_2_Te is a potential candidate for thermoelectric devices and Ultraviolet-shielding^8^. The phonon dispersion curve indicates neat stability and the further calculated parameters are firmly reliable. The alkali metal chalcogenides in its low dimensional structures have also gained more importance. The symmetry of the alkali metal chalcogenides is hexagonal or trigonal and can be stacked into layers. Li_2_S, Li_2_X (X = Se, Te), Na_2_X (X = S, Se) monolayers are predicted and studied by the researchers in very recent times^9–11^. These monolayers are indicated to be having promising applications at higher temperatures, in new nano-electronic, optoelectronic, and spintronic devices. The T-phase Rb_2_S and Rb_2_Se monolayers were predicted and examined via the first principles study, and may have photovoltaic and thermoelectric applications^12,13^. The analogous compounds such as SnS, SnSe, SnTe, TlSe, 2D hexagonal phases of Se and Te, SnI_2_, SiI_2_, BaX and AlX (X = S, Se, Te) monolayers and so on have discerning applications in the fields of thermoelectrics and optoelectronics^14–20^. Recent research in two-dimensional materials succeeded in investigating the hexagonal beryllium telluride (*h*-BeTe) monolayer via DFT. It has the figure of merit value of 0.9 at 300 K and displays good responsiveness in the UV–Vis regime of the electromagnetic spectrum^21^. Other research progressiveness based on 2D chalcogenides includes the findings of 2D Van der Waals heterostructures (XY_2_, X = Mo, W; Y = S, Se) with profound applications in photovoltaics and 2D tetragonal transition metal chalcogenides exhibits high performance in oxygen evolution and reduction areas^22–24^. Based on the theoretical and experimental observations of similar compounds^25^ the expected outcomes of the Rb_2_Te monolayer are to result in good electronic, optical and thermal flexibility. This stream of work is to investigate the ground state properties of the predicted dirubidium-telluride monolayer by employing the DFT. The electronic, optical, thermal and mechanical attributes of the monolayer are closely examined to understand the potential applications of the monolayer.

## Computational methodology

The density functional theory computational software “WIEN2k” aided for the fundamental understanding of the Rb_2_Te monolayer^26^. The unit cell structure was optimized and the calculations were performed via full potential linearly augmented plane wave (FP-LAPW) method. This geometry was deployed to comprehend the band gaps, density of states, charge bonding nature, optical and thermal response. The computations were done with the initial attributes of 12 × 12 × 1 k-mesh, *−7 Ryd* of energy cutoff, *10 L*_*max*_ and *R*_*mt*_*K*_*max*_ to be *7*, respectively. The PBE-GGA exchange correlation functional was employed for the computations of structural optimization and electronic structure calculations. To more precisely determine the electronic band gap value, the hybrid functional in WIEN2k, YS-PBE0 was used. The k-mesh of 12 × 12 × 1 was applied for these calculations. The dynamical stability was confirmed via Phonopy code interfaced with WIEN2k. It uses finite-displacement approach that involves the steps of generating force constants (force sets) and crystal symmetry. The choice of supercell for the phonon computations is 2 × 2 × 1. OPTIC code was run for the optical properties and the k-point mesh was increased to 24 × 24 × 1. BoltzTraP interface assisted in the determination of the thermal nature of the monolayer^27–31^. The results that stand by these input criterions are very well converged.

## Results and discussion

### Structural properties

**Fig. 1:**
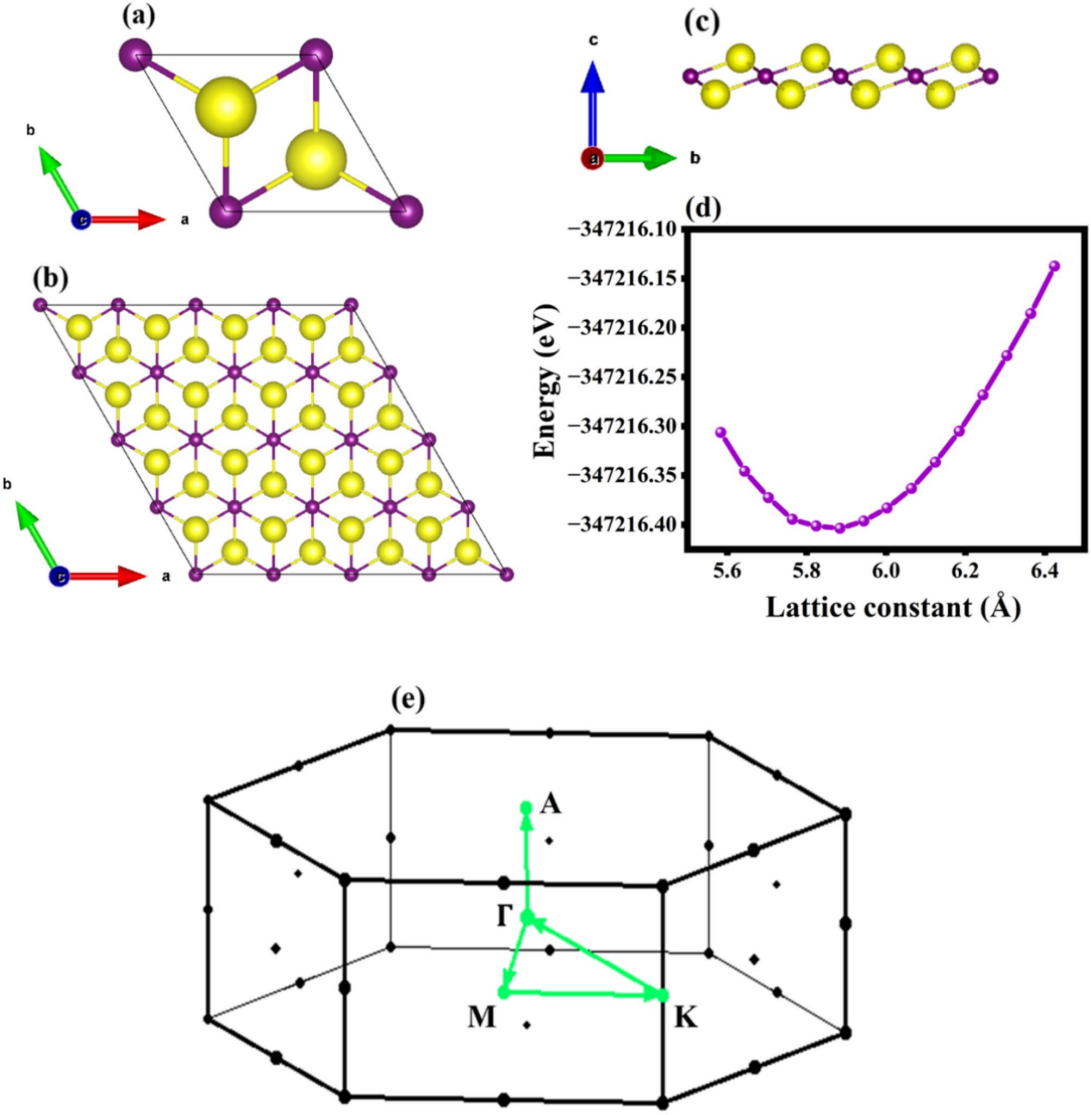
The optimized crystal structure of the monolayer. (**a**) Represents the unit cell of Rb_2_Te monolayer, (**b**), (**c**) are the top and side views of 4 × 4 × 1 supercell, (**d**) the optimization fit (lattice constant versus energy) and (**e**) Brillouin zone of Rb_2_Te monolayer.

One-atom thick layers are quite challenging to be exfoliated yet have exceptional continuum material properties. The di-rubidium telluride (Rb_2_Te) monolayer coalesces into the trigonal symmetry, in the space group 164. It resembles the centered honey-comb structures of Rb_2_S, Rb_2_Se and Rb_2_O with the chalcogen anions surrounding the alkali metal cations^13,14,32^. At first, the structure was optimized with the optimization curve shown in Fig. 1, the optimized lattice constants are a = b = 5.88 Å and the vacuum of c = 15 Å was extended along the z-axis. The top, all axial and side views of the monolayer in 1 × 1 × 1 unit cell and 4 × 4 × 1 supercell are displayed in Fig. 1a,b,c in which yellow atoms are Rb and violet atoms are Te, respectively. Being structurally stable, the electronic attributes were inferred via PBE-GGA exchange correlational potential. Figure 1d represents the optimization curve fit of Rb_2_Te monolayer and Fig. 1e is the Brillouin zone plot.

### Vibrational stability

**Fig. 2:**
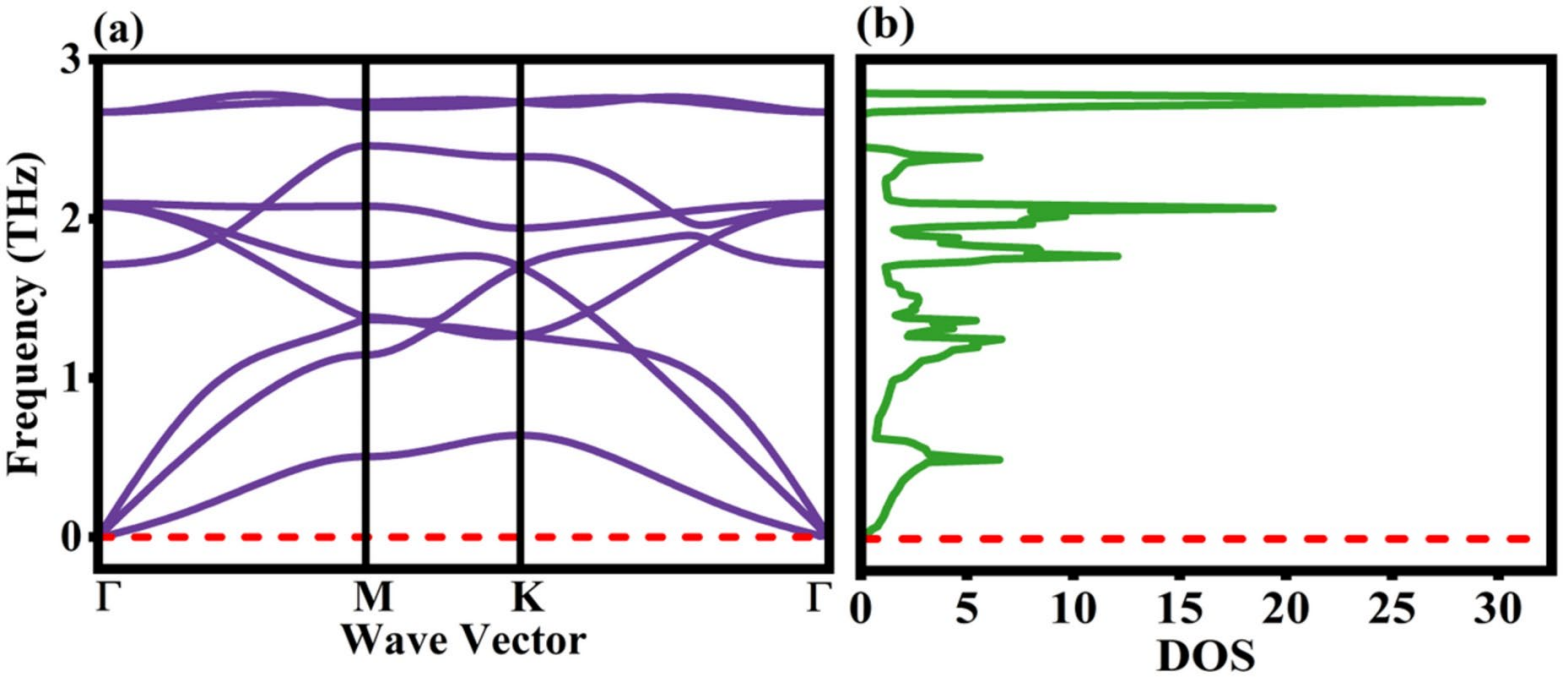
The vibrational stability with phonon characteristics of Rb_2_Te monolayer **(a)** Phonon dispersion curve plot and **(b)** DOS of phonon frequencies.

Phonons are the quantum of vibrational mechanical energy and they aid in the analysis of dynamical stability of a material. The excitations in a crystal are caused by the elementary particles, that are electrons and phonons. The electrical attributes are studied upon by the excitation of electrons and when the atoms oscillate within the crystal it results in the vibrational energy referred to as phonons, that are further used to analyze the acoustic and thermal properties of the crystal. There are two major phonon modes, acoustic and optical modes that have distinct levels of vibrational frequencies. The atomic simulations unified with the first principles approach is one of the promising models to predict and analyze the phonon behaviour in crystals and has evolved to design materials with phenomenal thermal properties^33^. The phonon dispersion curves give insights about the thermal properties (that aids in predicting thermal conductivities), electron–phonon interactions (which are crucial for electronic properties) and the dynamical stability of a material. The thermal conductivities can be inferred from the phonon dispersion curves. It is linked to the phonon group velocities which describes the speed of phonon wave packets through the material. The phonon energy is transferred faster in the acoustic branches due to high group velocities and the optical branches are flatter compared to the acoustic ones, hence they result in lower group velocities and have less contribution towards heat conduction. The Rb_2_Te monolayer has positive range of phonon frequencies over the Brillouin zones expanded with k-point mesh of 12 × 12 × 1 in the direction of $\Gamma$-M-K-$\Gamma$ and the force constants were generated for 2 × 2 × 1 supercell illustrated in Fig. 2a. Figure 2b depicts the density of occupied states w.r.t. the frequency. The phonon density of states (DOS) defines the number of phonon states available at each frequency level. The phonon distributions across distinct frequencies can be inferred from phonon DOS. Integrating the phonon dispersion curves over the entire Brillouin zone gives the phonon DOS and it reflects that the acoustic phonon modes contribute towards the lower frequency levels and optical phonon modes contribute towards the higher frequency levels. The phonon dispersion curves are advantageous in solving the Boltzmann transport equation in some advanced models^34^. These Brillouin zones are highly symmetrical and in total nine phonon branches were obtained that comprises of transverse (T) and longitudinal (L) branches with acoustic (TA, LA) and optical (TO, LO) modes. Therefore, the monolayer has three acoustic and six optical branches. The other phonon branches are known as flexural or out-of-plane acoustic (ZA) and optical (ZO) modes^35,36^. The positive spectrum of frequencies resembles that the monolayer is dynamically stable without any imaginary scales and the atoms will not rearrange to a lower energy state. The monolayers that belong to the similar series, such as Rb_2_S and Rb_2_Se are also dynamically stable^12,13^.

### Electronic properties

**Fig. 3:**
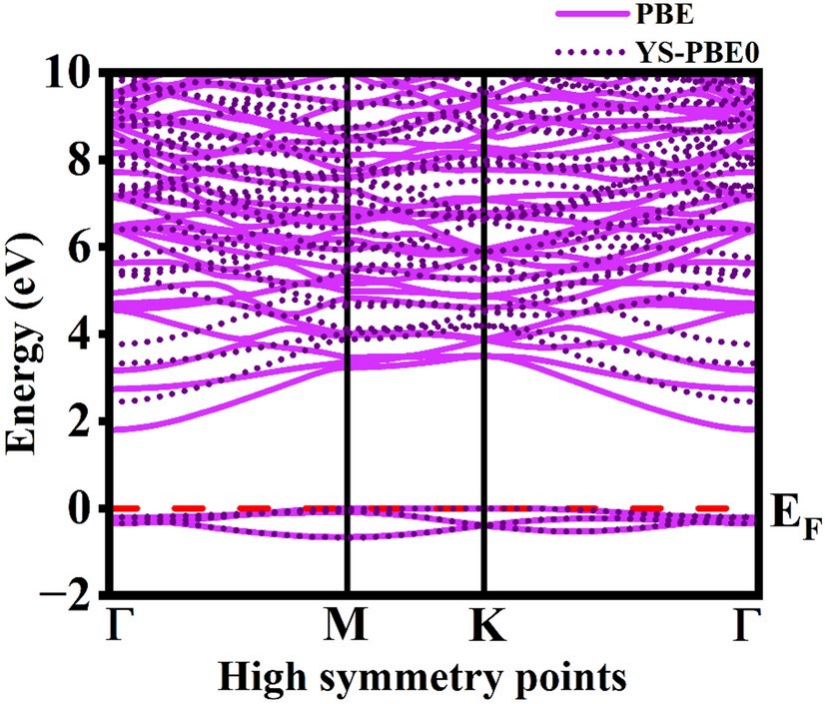
The electronic band structure of Rb_2_Te monolayer computed via PBE-GGA and YS-PBE0. The energy scale originated from the reference of setting fermi energy level to 0 eV.

**Fig. 4:**
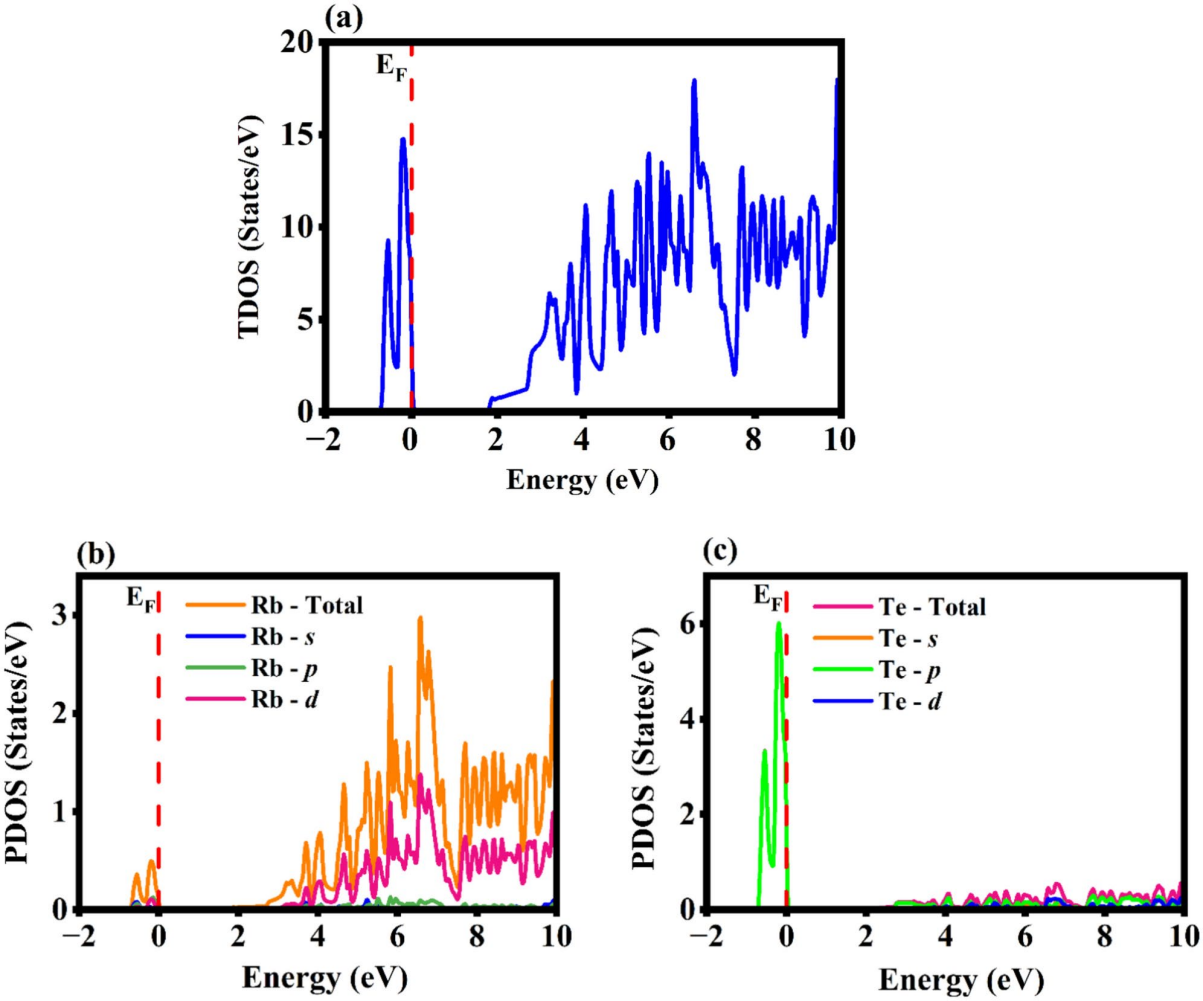
The density of states with each of the orbital contribution with the fermi energy level at 0 eV. (**a**) Depicts the TDOS profile, (**b**) and (**c**) represents the PDOS plots of Rb and Te, accordingly.

**Fig. 5:**
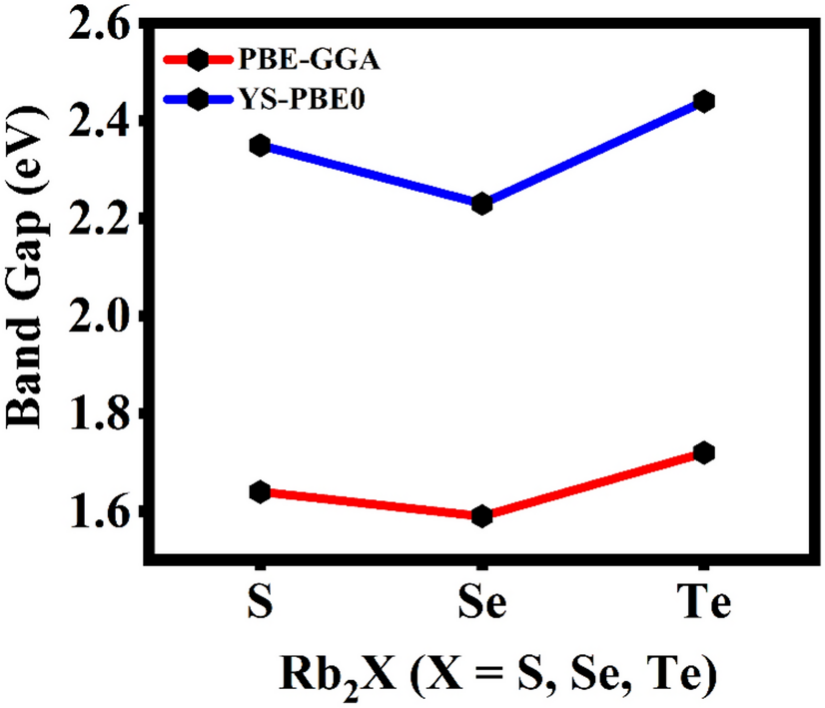
The band gaps of Rb_2_X (X = S, Se, Te) monolayers.

The energy band structure, density of states (total and partial DOS) that necessitates the contemplation of electronic parameters were encoded. The Brillouin zones in the reciprocal space were chosen from $\Gamma$ to $\Gamma$ that are high symmetry k-point vectors. The negative energy range is the valence band with holes and positive energy range is the unoccupied energy bands that will further constitute electrons during conduction. The fermi energy E_F_ is not attuned by any external force and is stationed at 0 eV. The $\Gamma$-M-K-$\Gamma$ symmetry was set in which the valence band maximum (VBM) and conduction band minimum (CBM) distinctly topples on M and $\Gamma$ k-points in the Brillouin zone. As the energy bands do not overlap, the monolayer has a semiconducting band nature with the energy band gap value of about 1.720 eV. For higher accuracy of the band gap value, the YS-PBE0 was employed from which the band gap was computed to be 2.44 eV. As VBM and CBM are misaligned at two different high symmetry k-points with respect to momentum, it is evidenced that the Rb_2_Te monolayer is an indirect band gap semiconductor represented in Fig. 3. The Table 1 depicts the Rb_2_X (X = S, Se, Te) monolayers lattice constant and band gap values reported via PBE-GGA. The lattice constant increases as the chalcogenide atomic number increases but the band gap value decreases and then increases for Rb_2_Te monolayer. The indirect band gap of Rb_2_Te monolayer is compared with similar indirect band gap monolayers such as Rb_2_S, Rb_2_Se and CdX (X = S, Se, Te) computed via PBESol with the corresponding values of 1.64, 1.59, 1.53, 1.03 and 0.77 eV respectively. The other monolayer that displays a similar band nature is PtS_2_ with the electronic band gap value of 1.79 eV computed via PBE^37^. The indirect band gap nature of the monolayer may display interesting applications in photovoltaics^38,39^. On applying the semiconducting statistics, the total number of states per unit area expressed as the function of energy is resolved. The DOS is the number of states at a given energy level that the electrons are allowed to occupy and projects how adjacent are the energy levels to each other. The two-dimensional materials have the motion confined along the z-axis with the degrees of freedom along x- and y-axis. In semiconductors, the spacing between the energy bands can be inferred via DOS. In the Rb_2_Te monolayer, the “no states” region is where there are no energy levels due to the indirect band gap (forbidden gap) of 1.720 eV represented in Fig. 4a. The PDOS gives insights about the elemental contribution towards the energy bands. The preponderant contribution of the orbitals of Rb and Te are represented in Fig. 4b,c respectively. The valence band (VB) is dominated by the *p*-orbital of Te and conduction band (CB) is dominated by the *d*-orbital of Rb. The *s*- and *p*-orbitals of Rb, and the *s*- and *d*-orbitals of Te have infinitesimal contribution towards the valence and conduction states. The conduction in the monolayer is apparently due to the *d*- and *p*-orbitals of the metal and the chalcogenide. The electronic band gap values computed via PBE-GGA and YS-PBE0 for the rubidium chalcogenide monolayers is represented in Fig. 5. The band gap value decreases till Se and increases for Te, this increase in the value is caused due to the interactions between *s*-*p* orbitals.

**Table 1:** The vital characteristics of Rb_2_X (X = S, Se, Te) monolayers.

Characteristics	Rb_2_S^12^	Rb_2_Se^13^	Rb_2_Te
Lattice constant (Å)	5.24	5.50	5.88
Electronic band gap (eV) PBE-GGA YS-PBE0	1.64 2.35	1.59 2.23	1.72 2.44

### Charge density

**Fig. 6:**
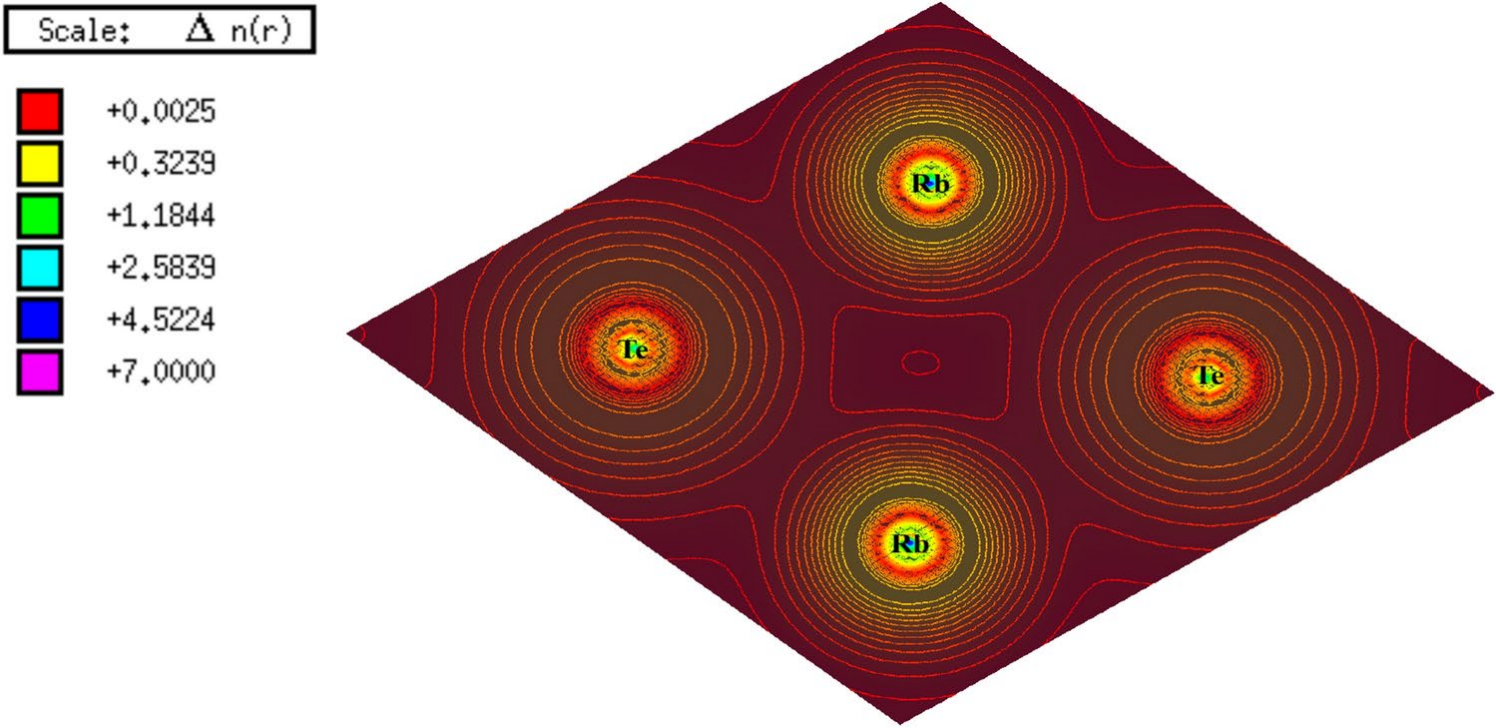
Surface charge density contour plot of Rb_2_Te monolayer with the charge density scale that represents the highest to lowest values of occupancy of the number of electrons per unit cell.

The surface charge density is the amount of charge per unit area in Cm^−2^ on a two-dimensional surface^40^. To comprehend the electronic charge density of the Rb_2_Te monolayer, three atoms spanning over the area were concealed and the contour plot is shown in Fig. 6. The surface charge density plot reveals that there is an ionic or electrovalent bond formation between the atoms of Rb and Te by the transfer of electrons. The charge density scale shows that there more electrons (highly intensive region) near to the nuclei and a smaller number of electrons in the interstitial region with less scale values.

### Elastic and mechanical properties

The determination of mechanical properties is another vital aspect of this work. The mechanical properties depend upon the elastic stiffness coefficients (C_ij_) and the Poisson’s ratio ($\nu$). The material’s response to the applied external forces can be unveiled by calculating these parameters. The mechanical stability of any material should satisfy the Born-Huang criterion given as,

$${C}_{11}\ge {C}_{12} \ge 0$$

$${C}_{66}= \frac{{C}_{11}- {C}_{12}}{2}\ge 0$$

The values of C_11_, C_12_ and C_66_ are given in Table 2. Positive elastic constants imply that the monolayer is suitable for practical applications. It has good withstanding ability of mechanical loads (stress, strain) and resists the structural deformations when subjected to different external strains (from − 2%, − 1%, 0%, 1% till 2%). The Rb_2_Se monolayer has good responsiveness to the applied external forces with positive elastic constants and Poisson’s ratio. `The layer modulus $(\gamma )$, shear modulus (K), in-plane stiffness constant (C) and the Poisson’s ratio are also examined. The Poisson’s ratio characterizes about the material’s change in perpendicular direction along the axis when stretched or compressed. The layer modulus is the measure of how the single-layer is resistant to deformation when the external force is applied. The $\gamma$, C and Pugh’s ratio are equated as,

1 $$\gamma = \frac{{C}_{11}+ {C}_{22}+2{C}_{12}}{4}$$

2 $$C= \frac{{C}_{11}^{2}-{C}_{12}^{2}}{{C}_{11}}$$

3 $$Pugh^{\prime}s\,ratio\, = \,\frac{\gamma }{K}$$

**Table 2:** The elastic coefficients C_11_, C_12_ and C_66_ in (GPa), the layer modulus $\gamma$ (GPa), shear modulus K (GPa), in-plane elastic stiffness constant C (GPa), Poisson’s ratio $\nu$ and Pugh’s ratio $\frac{\upgamma }{\text{K}}$ .

Monolayer	C_11_	C_12_	C_66_	${\varvec{\upgamma}}$	K	C	${\varvec{\upnu}}$	$\frac{{\varvec{\upgamma}}}{\mathbf{K}}$
Rb_2_Te	9.68	3.04	3.32	6.36	3.77	8.73	0.30	1.68

The Born-Huang criterion for the 1 T-Rb_2_Te monolayer is satisfied which makes the material mechanical stable. The lattice constant and the metal–chalcogen binding affects the layer modulus and in-plane stiffness values. The $\gamma$ is calculated to be 6.36 GPa and C is 8.73 GPa that can be compared to the other monolayers such as MoS_2_, graphene and NiX_2_ (X = O, S, Se) with the values of 136, 340, 137.64, 100.39 and 77.56 Nm^−1^ respectively^41^. The brittle and ductile materials can be identified from calculating the Pugh’s ratio. In some cases, it can also be obtained from the ratio of two mechanical properties (the shear modulus and the bulk modulus). If the $\frac{\gamma }{K}$ $\ge$ 1.75, the material displays ductile nature if not it is brittle. The $\frac{\gamma }{K}$ value of the monolayer is $<$ 1.75 that aids in confirming the brittle nature.

### Optical properties

**Fig. 7:**
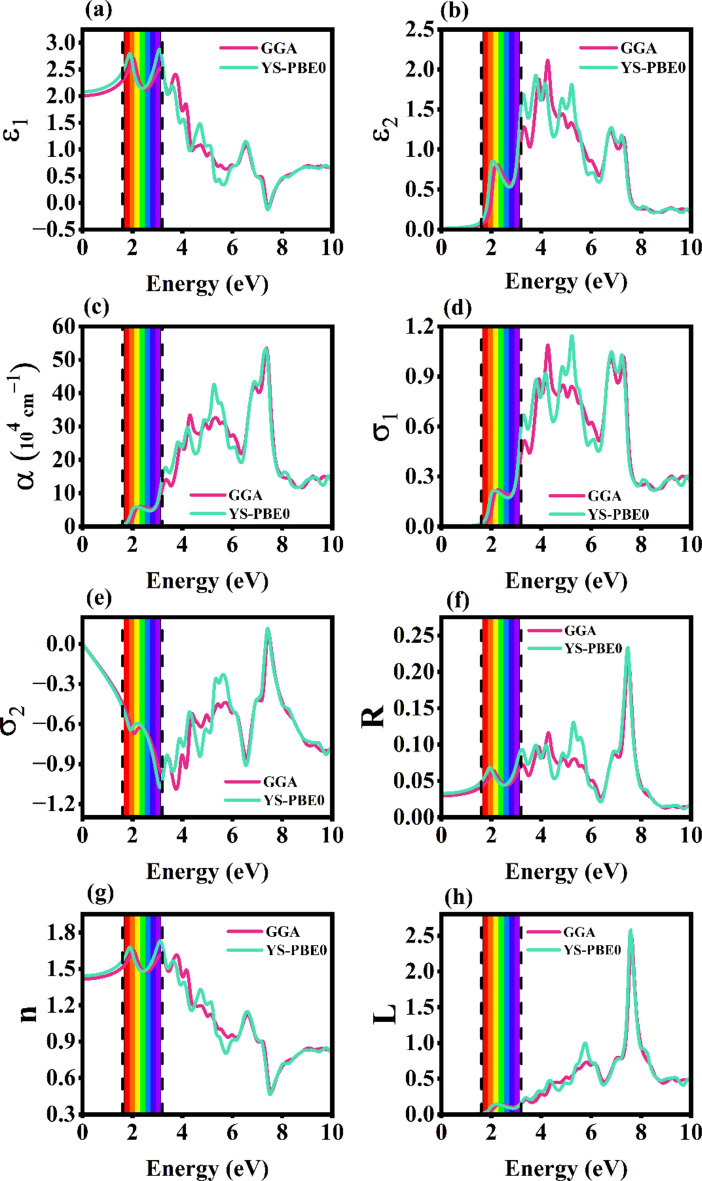
The optical responsiveness of Rb_2_Te monolayer computed via PBE-GGA and YS-PBE0. (**a**), (**b**) are real and imaginary dielectric functions, (**c**) is the optical absorption coefficient, (**d**), (**e**) are the real and imaginary optical conductivities, (**f**), (**g**), (**h**) are the reflectivity, refractive index, and energy loss profiles.

When the light (E = h $\nu$) strikes on a surface, some part of it is reflected by the surface, absorbed by the material, and the remaining amount is transmitted. These phenomena determine the visual appearance of a material. The sensitiveness of a material towards the electromagnetic radiation (mostly the visible region) can be inferred from examining its optical nature. Two of the exchange–correlation functionals, PBE-GGA and YS-PBE0 were deployed for better reliability of the optical results and are shown in Fig. 7. Mostly the optical characteristics are examined by PBE-GGA functional, the values obtained from it are well-reported in this section. The photon energy starts with the infrared region spanning from 0.0 to 1.7 eV, the visible region of the EM spectrum has the energy range from 1.6 to 3.2 eV and UV region covers the range from 3.1 to 12.6 eV, respectively. The materials that have neat and sharp peaks over 1.6 to 12.6 eV are regarded as good optoelectronic (direct band gap), photovoltaic (with decent band gap from 2.1 to 2.8 eV), and UV-based application materials^20,42,43^. The optical study of the monolayer is initiated upon by computing the dielectric function $\varepsilon (\omega )$ that is equated as,

4 $$\varepsilon \left(\omega \right)= {\varepsilon }_{1}(\omega )+ i{\varepsilon }_{2}(\omega )$$

The complex dielectric function, that describes the interaction between the electromagnetic spectrum and Rb_2_Te monolayer is the summation of the real ${\varepsilon }_{1}(\omega )$ and imaginary ${\varepsilon }_{2}(\omega )$ parts, in which the ${\varepsilon }_{1}(\omega )$ is linked to the refractive index and ${\varepsilon }_{2}(\omega )$ relies upon the optical absorption which is nearly related to the electronic band structure. They are computed upon by solving the following equations,

5 $${\varepsilon }_{1}\left(\omega \right)= Re\left(\varepsilon \left(\omega \right)\right)=1+ \frac{2}{\pi }P\underset{0}{\overset{\infty }{\int }}\frac{{\omega }{\prime}{\varepsilon }_{2}({\omega }{\prime})}{{\omega }^{{\prime}2}- {\omega }^{2}}{d\omega }{\prime}$$

6 $${\varepsilon }_{2}\left(\omega \right)= \left(\frac{4\pi {e}^{2}}{{\omega }^{2}{m}^{2}}\right)\sum_{ij}\int {\langle i\left|{M}_{cv}\right|j\rangle }^{2}{f}_{i}\left(1-{f}_{i}\right)*\delta \left({E}_{f}-{E}_{i}-\omega \right){d}^{3}k$$

The profile of ${\varepsilon }_{1}(\omega )$ as a function of energy is represented in the Fig. 7a. The static ${\varepsilon }_{1}(0)$ value is 2.00, the first and maximum peak is obtained at 2.02 eV with the value of ${\varepsilon }_{1}(\omega )$ as 2.71. The monolayer ideally possesses metallic nature with the negative ${\varepsilon }_{1}(\omega )$ values in the energy range of 7.38 to 7.52 eV. The imaginary spectra ${\varepsilon }_{2}(\omega )$ corresponding to the energy is depicted in the Fig. 7b. It has no profile in the infrared region and shows maximum of peaks from visible to UV range. The ${\varepsilon }_{2}(0)$ value is 0.01 and the first peak extents with 0.81 at 2.21 eV. The utmost point is obtained in the UV region at 4.25 eV. Once when the dielectric function is known the optical absorption $\alpha (\omega )$ can be derived from it via the formula,

7 $$\alpha \left(\omega \right)= \frac{\sqrt{2}}{c}\omega \sqrt{-{\varepsilon }_{1}\left(\omega \right)+\sqrt{{\varepsilon }_{1}\left({\omega }^{2}\right)+{\varepsilon }_{2}({\omega }^{2})}}$$

Figure 7c shows the optical absorption as function of energy profile of Rb_2_Te monolayer. It is correlated with the ${\varepsilon }_{2}(\omega )$ for crystalline materials and is governed by the features of electronic band structures. During the absorption of light, the peaks that are observed are similar to the ${\varepsilon }_{2}(\omega )$. In the infrared region there is no absorption, the absorption starts in the visible region after the electronic band gap with the static value of $\alpha (0)$ to be 6.20 × 10^4^ cm^−1^. The first peak of $\alpha (\omega )$ is at 2.29 eV with the value of 6.05 × 10^4^ cm^−1^ and ultimate value is obtained in the UV range with $\alpha (\omega )$ to be 53.54 × 10^4^ cm^−1^ at 7.36 eV. The higher absorption peaks obtained are due to inter-band transitions in the Rb_2_Te monolayer and are centered around the UV region. On comparing with Rb_2_S and Rb_2_Se monolayers, Rb_2_Te monolayer has maximum absorption peaks. Most of the UV electromagnetic radiation is absorbed by the monolayer. Figure 7d,e illustrates the optical conductivity profiles of its real ${\sigma }_{1}(\omega )$ and imaginary ${\sigma }_{2}(\omega )$ parts. The conducting ability of a material when light strikes on its surface can be inferred from these profiles. The electronic state of a material and its non-contact quantitative analysis are elaborately analyzed by knowing the optical conductivities^44^. ${\sigma }_{1}(0)$ and ${\sigma }_{2}(0)$ values are 2.09 × 10^15^ s^−1^ and -0.00 with each of its first and utmost values to be 0.22 × 10^15^ s^-1^ (2.27 eV), 1.08 × 10^15^ s^−1^ (4.25 eV) for ${\sigma }_{1}(\omega )$ -1.08 × 10^15^ s^−1^ (3.74 eV), −1.22 × 10^15^ s^−1^ (13.45 eV) for ${\sigma }_{2}(\omega )$ respectively. Reflectivity $R(\omega )$ determines the proportion of light reflected from a material’s surface and is the ratio of incoming luminous flux to the reflected luminous flux^45^. The Rb_2_Te monolayer’s $R(\omega )$ profile is shown in Fig. 7f where there is zero reflectivity at the static point and peaks in the UV region with 0.21 at 7.46 eV considering it for wave reflector applications. The Kramers–Kronig relations are linked to another vital parameter called refractive index $n(\omega )$. It is well-defined as the ratio of mobility/speed of light in a medium to the mobility/speed of light in vacuum. Light on passing via different mediums leads to the changes in its velocity and is majorly confirmed upon by estimating the $n(\omega )$ of the material. The Rb_2_Te monolayer has non-zero $n(0)$ that is 1.41 represented in the Fig. 7g and has the peak point in the visible range at 2.02 eV with the magnitude of 1.65. Energy loss $L(\omega )$ is an exceptional way to study the overall optical responsiveness of the compounds. The excitation of an electron in a solid can happen in various ways other than the absorption of photon energy. When a fast-moving electron passes in a solid, there might be losses in terms of energy that is referred to as eloss. The eloss spectra versus the energy of photons is shown in the Fig. 7h that has zero static value. The initial peak is at 2.35 eV and it spans in the UV regime and the maximum value is 2.48 at 7.60 eV. All the optical parameters adhere to the ultra-violet EM range, that makes the Rb_2_Te monolayer as a promising material in UV applications alike the beryllium chalcogenide monolayers (BeO, BeS, BeSe, BeTe) having UV related applications (UV-photodetectors and UV-protectant materials)^46–50^. The absorption coefficient value of Rb_2_Te > Rb_2_Se > Rb_2_S monolayer^12,13^. Table 3 represents the static, peak values of optical properties computed via PBE-GGA.

**Table 3:** Static and peak values of the optical properties of Rb_2_Te monolayer computed via PBE-GGA.

Rb_2_Te Monolayer		
Optical properties	Static values	Peak values (Energy eV)
${{\varvec{\varepsilon}}}_{1}$	2.00	2.71 (2.02)
${{\varvec{\varepsilon}}}_{2}$	0.01	2.11 (4.25)
$\boldsymbol{\alpha }$(10^4^ cm^−1^)	6.20 × 10^−4^	53.54 (7.36)
${{\varvec{\sigma}}}_{1}$(10^15^ s^−1^)	2.09 × 10^−5^	1.08 (4.25)
${{\varvec{\sigma}}}_{2}$(10^15^ s^−1^)	− 0.00	− 1.22 (13.45)
R	0.02	0.21 (7.46)
n	1.41	1.65 (2.02)
L	0.00	2.48 (7.60)

### Thermoelectric properties

Extrapolating the thermal properties of a material is essential for its applications towards thermoelectric devices. Renewable energy sources play a major role today as it gives a hand in reducing the global warming. The very best source of renewable energy is waste heat, that is converted into electricity through the Seebeck effect^51,52^. A magnificent or high-quality thermoelectric material should possess favorable figure of merit (ZT) value that depends upon the Seebeck coefficient (S), electrical conductivity ($\sigma )$ and temperature (T). The ZT is given by,

8 $$ZT= \frac{{S}^{2}\sigma T}{\kappa }$$

High Seebeck coefficient, electrical conductivity and low thermal conductivity values are required for a material to show good thermoelectric performance. There are many approaches that are being implemented to improve the ZT value. Decreasing the dimensionality of a material suffices in increasing the ZT (that is directly proportional to the Seebeck coefficient), helps in enhancing the energy conservation and storage. The S is linked to the density of states, larger the density of states near the Fermi level, larger is the S value.

The thermal parameters *S*, $\sigma$, $\kappa$, power factor and ZT versus the temperature T with the range of 100 to 1000 K are studied for the Rb_2_Te monolayer to unveil its thermoelectric applications. The Boltzmann transport equation is employed and solved via BoltzTraP code to study these thermal parameters. This code computes the relaxation-time $\tau$ (the $\tau$ value is fixed as 10^−14^ s), dependent electrical conductivity ($\sigma /\tau$) and $\tau$ dependent electronic thermal conductivity (${\kappa }_{e}/\tau$). In a semiconductor, the thermal conductivity comprises of electronic and thermal part due to electronic and phonon contribution. In the BoltzTraP code, lattice thermal conductivity ${\kappa }_{L}$ contributed by phonons is constant and it solves the ${\kappa }_{e}$ part alone. From the strong literature it can be said that the thermal conductivities are mainly due to the excited electrons in a semiconducting material and the lattice thermal conductivity (${\kappa }_{L}$) is inversely proportional to the temperature^53^. The (${\kappa }_{L}$) is calculated through the Slack equation which is discussed in brief below. The formulae involved in the computation of these properties are given as,

9 $${S}_{\alpha \beta }\left(T, \mu \right)= \frac{1}{eT\Omega {\sigma }_{\alpha \beta }(T, \mu )}\int {\sigma }_{\alpha \beta }\left(\epsilon \right)\left(\epsilon -\mu \right)\left[-\frac{\partial {f}_{0}(T, \epsilon , \mu )}{\partial \epsilon }\right]d\epsilon$$

10 $${\sigma }_{\alpha \beta }\left(T, \mu \right)= \frac{1}{\Omega } \int {\sigma }_{\alpha \beta }\left(\epsilon \right)\left[-\frac{\partial {f}_{0}\left(T, \epsilon , \mu \right)}{\partial \epsilon }\right]d\epsilon$$

11 $${\kappa }_{\alpha \beta }^{0}\left(T, \mu \right)= \frac{1}{{e}^{2}T\Omega }\int {\sigma }_{\alpha \beta }(\epsilon ){(\epsilon -\mu )}^{2}\left[-\frac{\partial {f}_{0}(T, \epsilon , \mu )}{\partial \epsilon }\right] d\epsilon$$

**Fig. 8:**
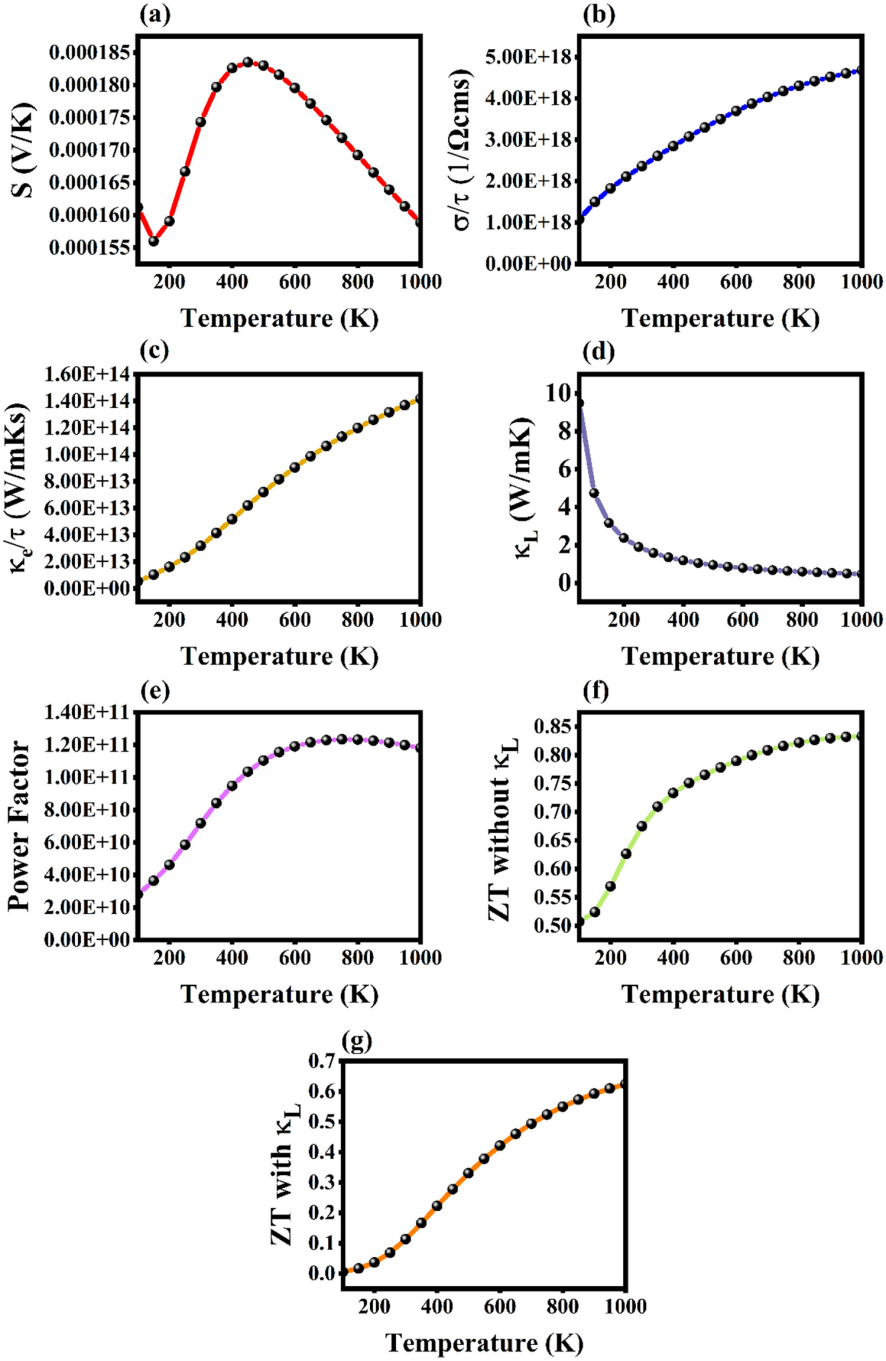
The thermoelectric flexibility of Rb_2_Te monolayer. (**a**) is the Seebeck coefficient, (**b**) represents electrical conductivity, (**c**) and (**d**) are the electronic and lattice thermal conductivities, (**e**) power factor, (**f**) and (**g**) are the ZT without ${\kappa }_{L}$ and ZT with ${\kappa }_{L}$.

The Seebeck coefficient *S*, for the Rb_2_Te monolayer at the room temperature is 174.33 $\mu$ VK^−1^ and gradually decreases as the temperature increases. At the lower temperatures there is an anomalous behavior in the *S* value that might be because of the flat bands near the fermi level, electron localization (due to quantum confinement effects in materials with low dimensionality), electron–electron and electron–phonon interactions. The type of semiconductor and dominant charge carriers can be confirmed from the *S* value. Positive *S* denotes p-type and negative *S* denotes n-type semiconductors. The *S* of Rb_2_Te is plotted in Fig. 8a that implies its positive type carriers or holes to be dominant and has a p-type semiconducting nature. Electrical conductivity $\sigma$, measures the flow of charge carriers from higher temperature to lower temperature regions that in turn generates electric current. The relaxation-time dependent electrical conductivity $\sigma /\tau$ is calculated for the Rb_2_Te monolayer and depicts a linear inclination with respect to temperature T. For semiconductors, the $\sigma /\tau$ values cognates with the electronic band structures. Figure 8b represents the linear curve of $\sigma /\tau$ with increasing temperature and depends on the negative temperature coefficient. At 300 K the $\sigma /\tau$ is 2.36 × 10^18^ $1/$ Ωcms and is compared to Rb_2_S, Rb_2_Se, GaX (X = S, Se, Te) monolayers^12,13,54^. The heat conduction across a material is measured via the thermal conductivity $\kappa$, that comprises of electronic and thermal parts. The Wiedemann–Franz law $\frac{{\kappa }_{e}}{\sigma }=LT$ states the temperature dependence of electronic part of thermal conductivity with respect to $\tau$, that is used to determine the ${\kappa }_{e}/\tau$ of the monolayer. Figure 8c depicts that the ${\kappa }_{e}/\tau$ plot of the monolayer which gradually increases with increasing temperature. At the room temperature the ${\kappa }_{e}/\tau$ value is 3.19 × 10^13^ Wm^−1^ K^−1^ s^−1^. To precisely study the figure of merit the lattice part of thermal conductivity ${\kappa }_{L}$ is necessary as it plays a vital role in semiconductors. The Slack equation is deployed to examine the ${\kappa }_{L}$ which is formulated as,

12 $${\kappa }_{L}= \frac{A \overline{{M }_{a}}\delta {{\theta }_{e}}^{3}}{{\gamma }^{2}T{n}^{2/3}}$$

The physical constant A, Gruneisen parameter $\gamma$ and Debye temperature ${\theta }_{e}$ are 3.08 × 10^−8^, 1.82 and 115.87 K respectively. $\overline{{M }_{a}}$, $\delta$ and n are the average molecular weight, cubic root of average volume and total number of atoms in the unit cell of the monolayer. The ${\kappa }_{L}$ plot is shown in Fig. 8d, ${\kappa }_{L}$ at 300 K is 1.58 W/mK and gradually decreases with inclination in the temperature. ${\kappa }_{L}$ at 700 takes the value of 0.67 W/mK and has the smallest value of 0.47 W/mK at 1000 K. The power factor (PF) determines the potential or performance of a material to generate large voltages and currents^55^. It is the product of the square of Seebeck coefficient and electrical conductivity that is relaxation-time dependent $(PF= {S}^{2}\sigma /\tau )$. The PF of the Rb_2_Te monolayer is plotted in Fig. 8e which constantly increases with temperature till 700 K, later decreases, saturates at 900 K and above. The value of PF at room temperature is 7.18 × 10^10^ Wm^−1^ K^−2^ s^−1^ for the Rb_2_Te monolayer that potentially helps in the figure of merit. The computed figure of merit (ZT) of Rb_2_Te as a function of temperature is shown in Fig. 8f. The ZT (without ${\kappa }_{L}$) curve shows similar characteristic like PF and it is large at high temperatures. At 300 K the ZT is 0.67, which confirms its use in thermoelectric device applications. The bulk Rb_2_Te has the ZT value of 0.03 at 300 K^8^ which is low compared to its monolayer at 300 K, this ensures that there is increase in ZT with decrease in dimensionality. It is requisite to compare the Rb_2_Te monolayer’s ZT values with similar compounds for the surety of its applications. The similar monolayers with their corresponding ZT values at 300 K such as Rb_2_S (0.55), Rb_2_Se (0.62), GaS (0.89), GaSe (0.90), GaTe (0.85), PdSe_2_ (ZT ranges from 0.5 to 1.1 based on the carrier concentration and doping effects), BaO (ZT > 0.25), BaSe (ZT > 0.85), BaTe (ZT > 0.75) and PbX (X = S, Se, Te with ZT > 1) have promising applications in thermoelectric device technologies^12,13,52–56^. As previously reported that at room temperature, the GaTe monolayer has the ZT value equal to 0.85 and below 300 K the ZT value is even more high, by this it is reported to have applications in low-temperature thermoelectrics. In the series of rubidium chalcogenide monolayers, the ZT of Rb_2_Te > Rb_2_Se > Rb_2_S. The Table 4. represents the thermoelectric ZT values of analogous compounds to Rb_2_Te monolayer. The ZT (with ${\kappa }_{L}$) at 30 , 700 and 1000 K is 0.11, 0.49, 0.62 illustrated in Fig. 8g proving its applications in waste heat recovery systems.

**Table 4:** ZT (without ${\kappa }_{L}$) values of series of Rubidium Chalcogenide Monolayers at room temperature (K).

Property	Rb_2_S^12^	Rb_2_Se^13^	Rb_2_Te
Figure of merit	0.55	0.62	0.67

### Thermodynamic properties

**Fig. 9:**
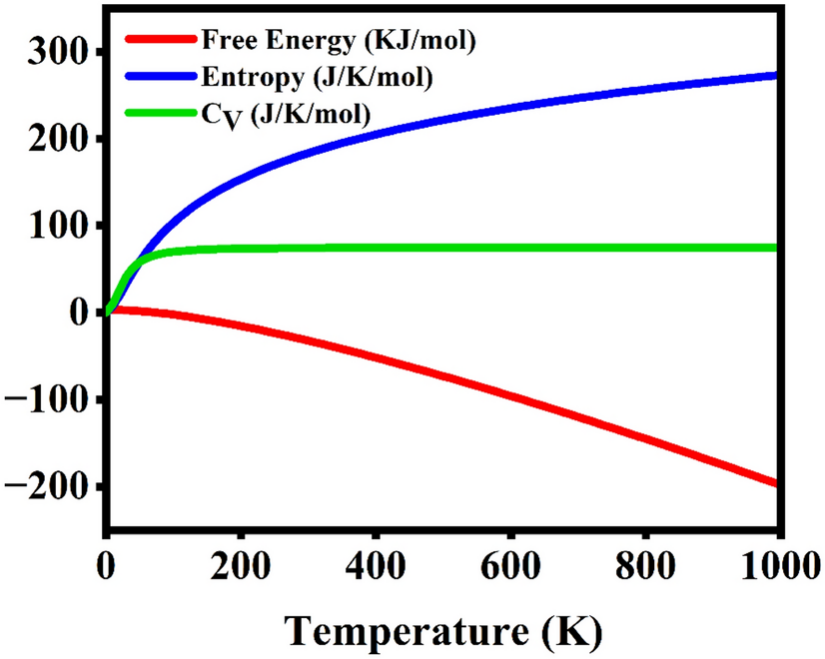
The thermodynamic properties of 1 T-Rb_2_Te monolayer verified from the properties of phonon vibrations.

The thermodynamic parameters such as free energy (F), entropy (s) and specific heat capacity at constant volume (C_V_) were computationally determined. As stated in the thermodynamics third law, when the temperature nears to absolute zero the entropy of the system remains constant, in Fig. 9 as the temperature increases gradually there is increase in entropy, heat capacity and decrease in free energy of the system that adheres to the thermodynamics third law^57^. Considering the Debye model and Dulong-Petit rule, the specific heat capacity increases till 150 K and is saturated at the higher temperatures that coheres to the laws.

### Effective mass

The effective mass (m^*^) theory of charge carriers depends on the band curvatures of CBM and VBM and is inversely proportional the charge carrier mobility. The excitonic effects are determined by the effective masses of electrons and holes in semiconductors^58^. The m^*^ of electrons and holes for the Rb_2_Te monolayer is estimated by fitting the parabolic curves of CBM and VBM accordingly (calculated by the fitting energy versus k-points plot). The components required for the determination of m^*^ are the energy band curvatures with the polynomial fitting. The reciprocal of the second order derivative times ${\hslash }^{2}$ (reduced Planck’s constant) gives the m^*^, i.e.,

13 $${m}^{*}= {\hslash }^{2}{\left(\frac{{d}^{2}E}{d{k}^{2}}\right)}^{-1}$$

where E and k are energy and wave vector values. The charge transfer mechanism is regulated by the effective masses and is inversely proportional to it. The recombination rate of the charge carriers can be deduced from the relative ratio (D = ${m}_{h}^{*}/{m}_{e}^{*}$) of the effective masses of holes and electrons. The rate of recombination is largely restrained due to very high D ratio and is high in Rb_2_Te than Rb_2_Se and Rb_2_S monolayers^12,13^. The Table 5 shows the electron, hole effective masses for Rb_2_Te monolayer.

**Table 5:** Effective masses, relative ratio of electrons and holes of Rb_2_Te monolayer.

Monolayer	Electrons	Holes	D
Rb_2_Te	0.30	6.05	20.16

## Conclusion

The Rb_2_Te monolayer was designed based on the analogous series of compounds via first principles calculations in the WIEN2k software. The physical properties such as structural, electronic, optical, and thermoelectric were concisely examined by DFT. The study aided in finding that the monolayer possesses T-phase geometrical structure with optimized in-plane lattice constant as a = b = 5.88 Å. The positive phonon modes and phonon DOS validates the vibrationally stability. It has an indirect band gap of 1.72 eV, that is furthermore electronically stable. The charge density contour elaborates that the monolayer is an ionic material. Positive elastic parameters aids in confirming the mechanical stability of the monolayer that shows brittle nature. Well-examined optical parameters from dielectric constants to the energy losses have maxima values in UV range and the absorption profile indicates higher values in UV regime than the similar rubidium chalcogenide monolayers. Thermoelectric characteristics apprises about the need of a good thermoelectric material on understanding the factors from Seebeck coefficient to the figure of merit. The ZT of Rb_2_Te monolayer is 0.67 at 300 K, that makes the monolayer a good choice of material in thermoelectric devices. On adhering to the thermodynamic laws, the single layer is also mechanically stable with holes as preponderant charge carriers. With righteous, favorable, and increasing material properties compared to the similar monolayers of rubidium chalcogenides, the Rb_2_Te can be a productive and suitable monolayer for UV and thermoelectric applications.

## Data Availability

The datasets used and/or analysed during the current study available from the corresponding author on reasonable request.

## References

[1] Novoselov, K. S. et al. Electric field effect in atomically thin carbon films. *Science* **306**(5696), 666–669. 10.1126/science.1102896 (2004). 15499015 10.1126/science.1102896

[2] Lebegue, S. & Eriksson, O. Electronic structure of two-dimensional crystals from ab initio theory. *Phys. Rev. B* **79**(11), 115409. 10.1103/PhysRevB.79.115409 (2009).

[3] Watanabe, K., Taniguchi, T. & Kanda, H. Direct-bandgap properties and evidence for ultraviolet lasing of hexagonal boron nitride single crystal. *Nat. Mater.* **3**(6), 404–409. 10.1038/nmat1134 (2004). 15156198 10.1038/nmat1134

[4] Alay-e-Abbas, S. M. & Shaukat, A. First principles study of structural, electronic and optical properties of polymorphic forms of Rb_2_Te. *Solid State Sci.* **13**(5), 1052–1059. 10.1016/j.solidstatesciences.2011.01.021 (2011).

[5] Bidai, K. et al. Structural, mechanical and thermodynamic properties under pressure effect of rubidium telluride: first principle calculations. *Arch. Metall. Mater.*10.1515/amm-2017-0127 (2017).

[6] Eithiraj, R. D., Jaiganesh, G. & Kalpana, G. First-principles study of electronic structure and ground-state properties of alkali-metal selenides and tellurides (M_2_A) [M: Li, Na, K; A: Se, Te]. *Int. J. Mod. Phys. B* **23**(25), 5027–5037. 10.1142/S0217979209052418 (2009).

[7] Souadia, Z., Bouhemadou, A., Khenata, R. & Al-Douri, Y. Structural, elastic and lattice dynamical properties of the alkali metal tellurides: First-principles study. *Phys. B Condens. Matter* **521**, 204–214. 10.1016/j.physb.2017.07.004 (2017).

[8] Khattak, S. A. et al. Ab-initio investigation of structural, optoelectronic, and transport properties of metal-alkali-based binary chalcogenides, X_2_Te [X= Na, K, Rb]: Rb_2_Te a potential candidate for UV-shielding and thermoelectric devices. *J. Mater. Res.* **38**(9), 2534–2549. 10.1557/s43578-023-00986-y (2023).

[9] Naseri, M. & Lin, S. 2D Li_2_S monolayer: A global minimum lithium sulfide sandwich. *Chem. Phys. Lett.* **722**, 58–63. 10.1016/j.cplett.2019.02.047 (2019).

[10] Bui, P. T., Nguyen, D. K., Guerrero-Sanchez, J. & Hoat, D. M. Exploration of new direct gap semiconductor Na_2_X (X= S and Se) monolayers. *Appl. Surf. Sci.* **606**, 154809. 10.1016/j.apsusc.2022.154809 (2022).

[11] Nguyen, D. K., Guerrero-Sanchez, J. & Hoat, D. M. Introducing the 1H-Na_2_S monolayer as a new direct gap semiconductor with feature-rich electronic and magnetic properties. *Phys. Chem. Chem. Phys.* **24**(44), 27505–27514. 10.1039/D2CP04613J (2022). 36342470 10.1039/d2cp04613j

[12] Sneha, G., Rueshwin, S. C. T. & Eithiraj, R. D. Structural, electronic, optical, and thermoelectric properties of 2D honeycomb-like 1T-Rb_2_S monolayer: A DFT study. *J. Phys. Chem. Solids* **181**, 111560. 10.1016/j.jpcs.2023.111560 (2023).

[13] Sneha, G. & Eithiraj, R. D. Combined DFT and MD simulation approach for the investigation of intrinsic material properties of T-phase Rb_2_Se monolayer. *J. Phys. Chem. Solids* **190**, 112020. 10.1016/j.jpcs.2024.112020 (2024).

[14] Fang, W. et al. Monolayer SnX (X= O, S, Se): two-dimensional materials with low lattice thermal conductivities and high thermoelectric figures of merit. *ACS Appl. Energy Mater.* **5**(6), 7802–7812. 10.1021/acsaem.2c01284 (2022).

[15] Majumdar, A., Chowdhury, S. & Ahuja, R. Ultralow thermal conductivity and high thermoelectric figure of merit in two-dimensional thallium selenide. *ACS Appl. Energy Mater.* **3**(9), 9315–9325. 10.1021/acsaem.0c01658 (2020).

[16] Ramírez-Montes, L., López-Pérez, W., González-Hernández, R. & Pinilla, C. Large thermoelectric figure of merit in hexagonal phase of 2D selenium and tellurium. *Int. J. Quantum Chem.* **120**(17), e26267. 10.1002/qua.26267 (2020).

[17] Betal, A., Bera, J. & Sahu, S. Low-temperature thermoelectric behavior and impressive optoelectronic properties of two-dimensional XI_2_ (X= Sn, Si): A first principle study. *Comput. Mater. Sci.* **186**, 109977. 10.1016/j.commatsci.2020.109977 (2021).

[18] Kumar, P., Rajput, K. & Roy, D. R. Structural, electronic, vibrational, mechanical, and thermoelectric properties of 2D and bulk BaX (X= O, S, Se and Te) series under DFT and BTE framework. *Phys. E Low Dimens. Syst. Nanostruct.* **127**, 114523. 10.1016/j.physe.2020.114523 (2021).

[19] Chen, X., Huang, Y., Liu, J., Yuan, H. & Chen, H. Thermoelectric performance of two-dimensional AlX (X= S, Se, Te): A first-principles-based transport study. *ACS Omega* **4**(18), 17773–17781. 10.1021/acsomega.9b02235 (2019). 31681883 10.1021/acsomega.9b02235 PMC6822128

[20] Bernardi, M., Ataca, C., Palummo, M. & Grossman, J. C. Optical and electronic properties of two-dimensional layered materials. *Nanophotonics* **6**(2), 479–493. 10.1515/nanoph-2015-0030 (2017).

[21] Makavana, A., Gajjar, J., Kumar, P. & Roy, D. R. Structural, electronic, thermoelectric and optical properties of 2D hexagonal beryllium telluride under DFT investigation. *Comput. Condens. Matter* **40**, e00948. 10.1016/j.cocom.2024.e00948 (2024).

[22] Dahbi, S., Ghaithan, H. M., Alkadi, M., Ahmed, A. A. A. & Qaid, S. M. Two-dimensional Van der Waals heterostructures based chalcogenide for photovoltaic applications: a DFT study. *Opt. Quantum Electron.* **56**(4), 507. 10.1007/s11082-023-06114-8 (2024).

[23] Cheng, Y. & Zhou, J. Two-dimensional tetragonal transition metal chalcogenides for high performance oxygen evolution and reduction: A DFT study. *ChemPhysChem* **25**(2), e202300714. 10.1002/cphc.202300714 (2024). 38010568 10.1002/cphc.202300714

[24] Bassman Oftelie, L. et al. Active learning for accelerated design of layered materials. *npj Comput. Mater.* **4**(1), 74. 10.1038/s41524-018-0129-0 (2018).

[25] Wang, V. et al. High-throughput computational screening of two-dimensional semiconductors. *J. Phys. Chem. Lett.* **13**(50), 11581–11594. 10.1021/acs.jpclett.2c02972 (2022). 36480578 10.1021/acs.jpclett.2c02972

[26] Blaha, P., Schwarz, K., Madsen, G.K., Kvasnicka, D. and Luitz, J. WIEN2k. An augmented plane wave+ local orbitals program for calculating crystal properties, 60 (1). (2001).

[27] Perdew, J. P., Burke, K. & Ernzerhof, M. Generalized gradient approximation made simple. *Phys. Rev. Lett.* **77**(18), 3865. 10.1103/PhysRevLett.77.3865 (1996). 10062328 10.1103/PhysRevLett.77.3865

[28] Monkhorst, H. J. & Pack, J. D. Special points for Brillouin-zone integrations. *Phys. Rev. B* **13**(12), 5188. 10.1103/PhysRevB.13.5188 (1976).

[29] Ambrosch-Draxl, C. & Sofo, J. O. Linear optical properties of solids within the full-potential linearized augmented planewave method. *Comput. Phys. Commun.* **175**(1), 1–14. 10.1016/j.cpc.2006.03.005 (2006).

[30] Madsen, G. K. & Singh, D. J. BoltzTraP. A code for calculating band-structure dependent quantities. *Comput. Phys. Commun.*10.1016/j.cpc.2006.03.007 (2006).

[31] Togo, A. & Tanaka, I. First principles phonon calculations in materials science. *Scr. Mater.* **108**, 1–5. 10.1016/j.scriptamat.2015.07.021 (2015).

[32] Rueshwin, S. C. T. & Eithiraj, R. D. An investigation of the 2D Rb_2_O monolayer intrinsic properties from a theoretical perspective using DFT. *J. Phys. Chem. Solids* **187**, 111855. 10.1016/j.jpcs.2023.111855 (2024).

[33] Gu, X. & Yang, R. Phonon transport and thermal conductivity in two-dimensional materials. *Annu. Rev. Heat Transf.*10.1615/AnnualRevHeatTransfer.2016015491 (2016).

[34] Peng, B. et al. Phonon transport properties of two-dimensional group-IV materials from ab initio calculations. *Phys. Rev. B* **94**(24), 245420. 10.1103/PhysRevB.94.245420 (2016).

[35] Bao, D. L. et al. Phonon vortices at heavy impurities in two-dimensional materials. *Nanoscale Horiz.* **9**(2), 248–253. 10.1039/D3NH00433C (2024). 38091005 10.1039/d3nh00433c

[36] Mounet, N. & Marzari, N. First-principles determination of the structural, vibrational and thermodynamic properties of diamond, graphite, and derivatives. *Phys. Rev. B* **71**(20), 205214. 10.1103/PhysRevB.71.205214 (2005).

[37] Liang, X. C., He, X. J., Ding, Y. C., Hao, Y. J. & Zhu, J. Transport properties and thermoelectric properties of two-dimensional PtS_2_ monolayer: First-principle study. *Phys. E Low Dimens. Syst. Nanostruct.* **132**, 114744 (2021).

[38] Naseri, M., Hoat, D. M., Rivas-Silva, J. F. & Cocoletzi, G. H. Electronic structure, optical and thermoelectric properties of cadmium chalcogenides monolayers. *Optik* **210**, 164567. 10.1016/j.ijleo.2020.164567 (2020).

[39] Mogulkoc, Y., Modarresi, M., Mogulkoc, A., Ciftci, Y. O. & Alkan, B. First principle and tight-binding study of strained SnC. *J. Phys. Chem. Solids* **111**, 458–463. 10.1016/j.jpcs.2017.08.036 (2017).

[40] Vijay, A., Hariharan, M., Sneha, G. & Eithiraj, R. D. Wannier-Mott excitons in alkali metal-based nitridorhenate: A DFT study. *Mater. Today Commun.* **33**, 104952. 10.1016/j.mtcomm.2022.104952 (2022).

[41] Kumar, V., Kumar, R. & Chand, F. First principle calculations to explore the electronic, mechanical and optical properties of 2D NiX_2_ (X= O, S, Se) monolayers. *Phys. B Condens. Matter* **686**, 416066. 10.1016/j.physb.2024.416066 (2024).

[42] Ermolaev, G. A. et al. Broadband optical properties of monolayer and bulk MoS_2_. *npj 2D Mater. Appl.*10.1038/s41699-020-0155-x (2020).

[43] Garcés, E., Salas, O. & Magaña, L. F. Optical absorption and reflectivity of four 2D materials: MoS_2_, MoP_2_, NbS_2_, and NbP_2_. *Front. Mater.* **8**, 720768. 10.3389/fmats.2021.720768 (2021).

[44] Bai, Y. et al. Dependence of elastic and optical properties on surface terminated groups in two-dimensional MXene monolayers: a first-principles study. *RSC Adv.* **6**(42), 35731–35739. 10.1039/C6RA03090D (2016).

[45] Barhoumi, M., Said, I., Sfina, N., Al-Saleem, N. K. & Ghrib, T. A DFT study of the electronic and optical properties of four 2D thin films. *Mater. Chem. Phys.* **286**, 126158. 10.1016/j.matchemphys.2022.126158 (2022).

[46] Wang, S. F. & Wu, X. J. First-principles study on electronic and optical properties of graphene-like boron phosphide sheets. *Chin. J. Chem. Phys.* **28**(5), 588–594. 10.1063/1674-0068/28/cjcp1505100 (2015).

[47] Kumar, P., Rajput, K. & Roy, D. R. First principle investigation on 2D beryllium chalcogenides for thermoelectric and optical applications. *J. Phys. Chem. Solids* **164**, 110619. 10.1016/j.jpcs.2022.110619 (2022).

[48] Sang, D. K. et al. Electronic and optical properties of two-dimensional tellurene: from first-principles calculations. *Nanomaterials* **9**(8), 1075. 10.3390/nano9081075 (2019). 31357462 10.3390/nano9081075 PMC6722590

[49] Bhattarai, R., Chen, J., Hoang, T. B., Cui, J. & Shen, X. Anisotropic optical properties of 2D silicon telluride. *MRS Adv.* **5**(35–36), 1881–1889. 10.48550/arXiv.1906.11225 (2020).

[50] Li, Z., Yao, F. & Sun, H. Reinforced active learning for CVD-grown two-dimensional materials characterization. *IISE Trans.* **56**(8), 811–823. 10.1080/24725854.2023.2227659 (2024).

[51] Shafique, A. & Shin, Y. H. Thermoelectric and phonon transport properties of two-dimensional IV–VI compounds. *Sci. Rep.* **7**(1), 506. 10.1038/s41598-017-00598-7 (2017). 28360412 10.1038/s41598-017-00598-7 PMC5428725

[52] Haq, B. U. et al. Exploring the potential of lead-chalcogenide monolayers for room-temperature thermoelectric applications. *Ceram. Int.* **47**(3), 3380–3388. 10.1016/j.ceramint.2020.09.183 (2021).

[53] Bahuguna, B. P., Saini, L. K., Sharma, R. O. & Tiwari, B. Hybrid functional calculations of electronic and thermoelectric properties of GaS, GaSe, and GaTe monolayers. *Phys. Chem. Chem. Phys.* **20**(45), 28575–28582. 10.1039/C8CP04723E (2018). 30403246 10.1039/c8cp04723e

[54] Qin, D. et al. Monolayer PdSe_2_: A promising two-dimensional thermoelectric material. *Sci. Rep.* **8**(1), 2764. 10.1038/s41598-018-20918-9 (2018). 29426886 10.1038/s41598-018-20918-9 PMC5807448

[55] Wang, H. et al. First-principles study of electronic, optical and thermal transport properties of group III–VI monolayer MX (M= Ga, In; X= S, Se). *J. Appl. Phys.*10.1063/1.5094663 (2019).32189721

[56] Chen, E., Xu, W., Chen, J. & Warner, J. H. 2D layered noble metal dichalcogenides (Pt, Pd, Se, S) for electronics and energy applications. *Mater. Today Adv.* **7**, 100076. 10.1016/j.mtadv.2020.100076 (2020).

[57] Hariharan, M. & Eithiraj, R. D. Investigations on the thermoelectric and thermodynamic properties of quaternary coinage metal HgSBr. *Heliyon*10.1016/j.heliyon.2023.e19438 (2023). 37810057 10.1016/j.heliyon.2023.e19438 PMC10558493

[58] Huang, L., Chen, Z. & Li, J. Effects of strain on the band gap and effective mass in two-dimensional monolayer GaX (X= S, Se, Te). *RSC Adv.* **5**(8), 5788–5794. 10.1039/C4RA12107D (2015).

